# Single-Incision Laparoscopic Surgeries for Colorectal Diseases: Early Experiences of a Novel Surgical Method

**DOI:** 10.1155/2012/783074

**Published:** 2012-07-19

**Authors:** Tomoki Makino, Jeffrey W. Milsom, Sang W. Lee

**Affiliations:** Division of Colon and Rectal Surgery, New York Presbyterian Hospital, Weill Cornell Medical College, 525 East 68th Street, Box 172, New York, NY 10021, USA

## Abstract

*Objectives*. This paper aims to analyze the feasibility and safety of single-incision laparoscopic colectomy (SILC) and its potential benefits. *Methods*. Systematic review was performed for the years 1983–August 2011 to retrieve all relevant literature. A total of 21 studies with 477 patients undergoing SILC were selected. *Results*. Range of operative times and estimated blood losses were 75–229 min and 0–100 mL, respectively. Overall conversion rate was 5.9% (28/477) and an additional laparoscopic port was used in 4.9% (16/329) cases. Range of lymph node number for malignant cases was 12–24.6 and surgical margins were all negative. Overall mortality and morbidity rate was 0.4% (2/477) and 11.7% (43/368), respectively. The length of hospital stay (LOS) varied across reports (2.7–9.2 days). Among 6 case-matched studies, one showed less blood loss in SILC as compared to LAC and 2 showed shorter LOS after SILC versus HALC or LAC/HALC groups. In addition, one study reported maximum pain score on postoperative days 1 and 2 was lower in SILS compared to LAC and HALC. *Conclusions*. SILC procedure is feasible and safe when performed by surgeons highly skilled in laparoscopy. In spite of technical difficulties, there may be potential benefits associated with SILC over LAC/HALC.

## 1. Introduction

Recently, laparoscopic surgeries have been widely accepted as a treatment of colon diseases including colon cancer [1–3]. Most surgeons are convinced by the short time benefit of the laparoscopic approach in colorectal surgery, that is, early postoperative recovery, decreased postoperative pain, reduced pulmonary dysfunction, and shorter hospitalization [4–6]. Moreover, in oncological terms, it has also been shown to be safe in the treatment of colon cancer [1, 2]. In order to further improve upon the results of multiport laparoscopic colectomies (LACs), efforts have been made to further reduce the trauma caused by incisions. The rationale for further “scar-less” surgery is that decreasing the number and size of port accesses to the abdominal cavity might be an advantage not only from the cosmetic aspect but also in minimizing the risk of complications such as wound pain and infections as well as incision hernia and internal adhesion formation [7].

The excitement to develop new techniques has given rise to natural orifice transluminal endoscopic surgery (NOTES) [8–10]. This procedure in both animal [11] and human [12] models has shown some success but certainly has technical challenges: using transgastric, transvaginal, and transrectal access to the abdominal viscera and the need for expensive specialized equipment has hindered the widespread acceptance of this approach. Therefore use of the NOTES approach in performing routine colon resection is far from being practical at this time. Single-incision laparoscopic surgery (SILS) has advantages over NOTES in that existing laparoscopic instruments can be used and relatively minor adjustments from the current multiport laparoscopic technique are needed. The initial applications of SILS in gastrointestinal surgery were cholecystectomy [13], appendectomy [14] and recently, this technique has also been applied to colorectal surgery [15–18].

**Figure 1:**
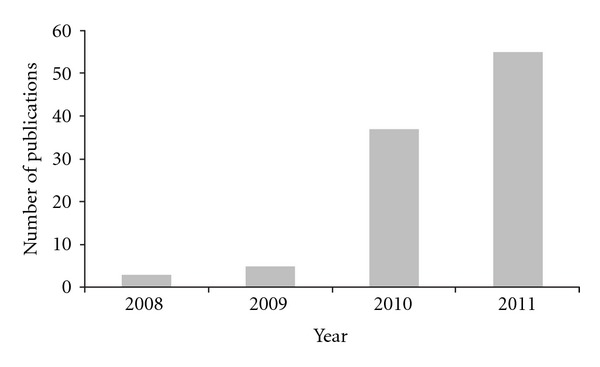
The number of publications regarding single-incision laparoscopic colectomy.

In comparison to multiport laparoscopic colectomy, the potential advantages of SILS are thought to be improved cosmesis as well as incisional and/or parietal pain and avoidance of port site-related complications [40]. Since 2008 when single-incision laparoscopic colectomy (SILC) was first introduced, the number of relevant publications has been increasing year by year as shown in Figure 1. However, because of still limited number of studies reporting SILC [41], its clinical significance remains to be elucidated. The aim of this study is to analyze current literature on SILC and access its potential benefits or efficacy as well as its feasibility and safety.

## 2. Materials and Methods

### 2.1. Literature Search Strategies

A systematic search of the scientific literature was carried out using the MEDLINE, EMBASE, the Cochrane Central Register of Controlled Trials ClinicalTrials.gov (Available at: http://clinicaltrials.gov/), National Research Register, The York (UK) Centre for Reviews, American College of Physicians (ACP) Journal Club, Australian Clinical Trials Registry, relevant online journals, and the Internet for the years 1983–August 2011 to obtain access to all relevant publications, especially randomized controlled trials, systematic reviews, and meta-analyses involving SILC. The search terms were “single-incision,” “single port,” “single access,” “single site,” “laparoscopic colectomy,” “colectomy,” and “laparoscopic colorectal surgery.”

### 2.2. Inclusion and Exclusion Criteria

**Figure 2:**
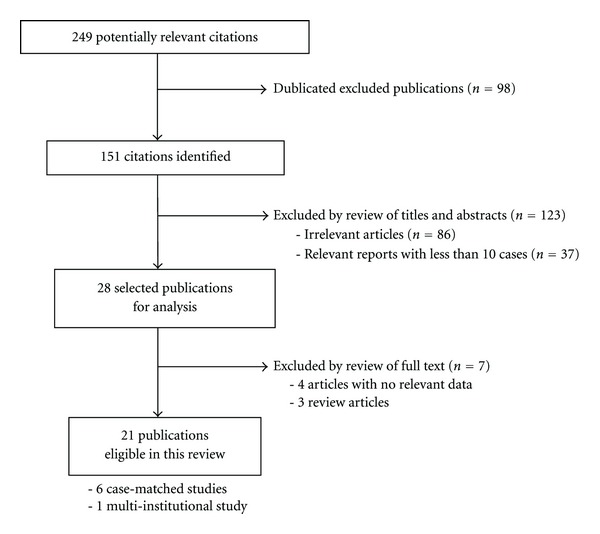
Flow chart of the selection process for studies included in the systematic review.

Articles were selected if the abstract contained data on patients who underwent SILC for colorectal diseases in the form of RCTs and other controlled or comparative studies. Conference abstracts were included if they contained relevant data. The reference lists of these articles were also reviewed to find additional candidate studies. Searches were conducted without language restriction. To avoid duplication of data, articles from the same unit or hospital were included only once if data was updated in a later publication. However, if surgical cases did not overlap among reports by even the same institute, these reports were all included. Reports with fewer than 10 cases of SILC and review articles were excluded from this study. Data extracted for this study were taken from the published reports; authors were not contacted to obtain additional information. All articles selected for full text review were distributed to 2 reviewers (T.M and S.L.), who independently decided on inclusion/exclusion and independently abstracted the study data. Any discrepancies in agreement were resolved by consensus. The flow chart of this selection process is summarized in Figure 2.

### 2.3. Result of the Literature Research

By using the above search strategy, a total of 249 potentially relevant citations were found. After the exception of 98 duplicated citations, we excluded 86 articles irrelevant of surgical specialty and 37 relevant articles with fewer than 10 cases by reviewing titles and abstracts. 28 publications were selected for review of full text, and 4 studies with no relevant data and 3 review articles were excluded from our paper. Twenty-one studies [19–39] with a total of 477 patients undergoing SILC met the criteria for analysis providing level 2–4 evidence (Table 1). There were one multi-institutional study and a total of 9 comparative studies including 6 case-matched ones between SILC and other minimally invasive procedures. There were no randomized controlled trials and meta-analyses in the selected literature.

## 3. Results

### 3.1. Indications and SILC Procedures

Demographic information and preoperative parameters are shown in Table 1. All studies except 4 performed SILC for colon cancer cases [21, 26, 29, 38]. Among them, 18 studies also included benign colon disease (diverticulitis, Crohn's disease, ulcerative colitis, polyps, etc.) [21, 22, 24–39]. The most common surgical procedures performed in these series were right hemicolectomy (*n* = 277), followed by sigmoidectomy (*n* = 81). Anterior resections were performed in 5 of 22 studies (*n* = 37). Range of body mass index (BMI) was 21.9–30.0 kg/m^2^ in each study.

### 3.2. Surgical Instruments and Skin Incision Length

All studies except one [30] used commercially available single port devices as summarized in Table 3. Chen et al. used a surgical glove attached with three trocars for the purpose of reestablishing the pneumoperitoneum after extraction of the specimen and anastomosis [30]. Ross et al., instead of a single access device, used multiple trocars placed through a single skin incision for some patients [32]. All studies, with exception of two [29, 34], utilized three ports/trocars (5, 5, 5, or 12 mm) placed through the single access device. Sixteen studies reported on type of laparoscope used [20–26, 29, 30, 32–38]. Most of investigators from the studies reported using 30°-angled scopes while two studies used 0° laparoscopes [20, 21]. Types of instruments used are detailed in Table 3. The skin incision for the insertion of port systems initially measured 2 to 4 cm, and average length of final scar was 2.7–4.5 cm in 7 studies [22, 23, 27, 31–33, 36] with relevant data. The final (at the end of operation) length of incision scar was longer than the initial one in all 11 studies with available data [21–24, 27, 28, 30, 33–36].

### 3.3. Intraoperative Parameters

The summary of various operative parameters is shown in Table 2. The range of operative times for SILC procedure was 75–229 minutes (*n* = 21 studies). The range of estimated blood loss was 0–100 mL (*n* = 14 studies). Among all 477 cases eligible in the current paper, a total of 5 cases (1.0%) were converted to open procedures, 3 cases (0.6%) to hand-assisted laparoscopic surgeries (HALS), and 20 cases (4.2%) to conventional (multiport) laparoscopic colectomies (LAC). Overall conversion rate was 5.9% (28/477). Reasons of conversion in these cases were the following: purpose for retraction or aid in colonic mobilization (*n* = 9), severe adhesion (*n* = 4), port trouble (*n* = 3), low-rectal lesions (*n* = 3), obesity (*n* = 3), bleeding (*n* = 1), fistula (*n* = 1), time constrains (*n* = 1), facilitating primary suture closure of colorectal anastomosis following a positive air insufflation test (*n* = 1), T4 tumor (*n* = 1), and unknown reason (*n* = 1). On the other hand, among 15 studies (*n* = 329) with available data, an additional port (adding only one port) was needed during the operation in a total of 16 cases (4.9%; 16/329). No major intraoperative complications were observed in these series.

### 3.4. Surgical Specimen

Five studies including right hemicolectomy, sigmoidectomy, and anterior resection showed that the range of specimen lengths was 15–43.5 cm (Table 4) [20, 24, 27, 28, 35]. All margins were free of cancer in these series. In 18 studies with available data, the range of number of removed lymph nodes for malignant cases and potential malignant diseases was 12–24.6 (Table 4) [19, 20, 22–25, 27, 28, 30–39].

### 3.5. Postoperative Parameters

#### 3.5.1. Perioperative Mortality

Overall, 2 perioperative deaths (0.4%; 2/477) were observed. One death, reported by Adair et al., occurred on postoperative day 10, 8 days after discharge from the hospital, due to a pulmonary embolus [36]. Gandhi et al. reported another death, which was encountered in a patient following palliative SILC right hemicolectomy as a result of complications from metastatic disease [33].

#### 3.5.2. Morbidity, Reoperation, and Length of Hospital Stay (LOS)

Postoperative morbidities varied across studies (0–29.4%). Overall 43 patients (11.7%; 43/368) developed complications related to surgery. The most frequent complication was ileus (*n* = 10) and wound infection/hematoma/seroma (*n* = 10) followed by and anastomotic bleeding (*n* = 4) and arrhythmia (*n* = 3). Overall 6 out of 419 patients (1.4%) required reoperation and the reasons in these cases were as follows: anastomotic leakage (*n* = 2), anastomotic bleeding (*n* = 1), wound hematoma (*n* = 1), cecal ischemia with perforation (*n* = 1), and a negative relaparotomy to rule out anastomotic leakage (*n* = 1). In all 21 studies, the range of length of hospital stay (LOS) also varied across reports: 2.7–9.2 days. Notably, 2 studies reported fewer than 3 days of LOS in their series [33, 37].

#### 3.5.3. Postoperative Anesthesia

Katsuno et al. reported that analgesics were used 1.4 ± 1.2 times in addition to routinely using the epidural catheter (0.2% ropivacaine hydrochloride hydrate 600 mg plus morphine hydrochloride hydrate 8 mg) for the first 2 to 3 days as postoperative anesthesia and no patients required analgesics after the fourth postoperative day [23]. Wolthuis et al. reported that total consumption of levobupivacaine (313 versus 355 mg) and sufentanyl (250 versus 284 *μ*g) provided by epidural infusion with a patients-controlled bolus capability was similar between SILC and LAC groups (*P* = 0.94) [24]. Chen et al. also found no difference in the postoperative usage of intravenous narcotics (Demerol) between SILC and LAC groups (10 versus 10 mg, *P* = 0.82) [30].

#### 3.5.4. Postoperative Recovery of Gastrointestinal Function

Several reports [21, 23, 26, 29, 30, 37, 39] provided data regarding postoperative recovery of gastrointestinal function; Gash et al. [37], in their analysis of 20 SILC procedures, reported that a normal diet was tolerated in 4–6 hours by 7 patients and in 12–16 hours (overnight) by 11 patients. In 39 SILC cases [32] from multi-institutional studies reviewed, average time to flatus and bowel movement were Days 2.2 and 2.9, respectively, which is supported by 2 other reports (p.o. Day 2-3 of first flatus) [21, 30, 42, 43]. Chen et al., in their case-control study comparing SILS right hemicolectomy to traditional laparoscopic right hemicolectomy, also reported that there was no difference in time until flatus passage (median 2 versus 2 days) [30]. Concerning oral intake after surgeries, Boni et al. [39] reported p.o. Day 2 for first oral fluid intake. In early experience with 31 SILC cases for colon cancer, Katsuno et al. reported that the time to adequate oral intake was 1.5 ± 0.8 days [23].

### 3.6. Comparative Studies: SILC versus Other Minimally Invasive Surgeries

A total of 9 comparative studies [19, 22, 24, 27, 30, 31, 33, 35, 36] including 6 case-matched studies [22, 24, 27, 31, 33, 36] between SILC and other minimally invasive procedures are summarized in Tables 5 and 6. Ramos-Valadez et al., in their case-matched series (SILC versus LAC group), reported that mean estimated blood loss was significantly lower for the SILC group (*n* = 20) compared to the LAC group (*n* = 20) (58 versus 99 mL, *P* < 0.007) [22]. Champagne et al., in their case-controlled study comparing SILC (*n* = 29) versus laparoscopic-assisted (*n* = 29) segmental colectomy, reported that SILC is feasible and safe but takes longer time in surgery (134 versus 104 min *P* = 0.0002) [27]. There were no short-term outcome benefits associated with SILC. Chen et al. also did not find any significant benefits associated with right hemicolectomy by SILS approach compared to the same procedure by the multiport laparoscopic approach [30]. McNally et al., comparing 27 SILC cases with 46 LAC cases, reported relatively shorter LOS in SILC versus LAC cases (3 versus 5 days) but with no statistical significance (*P* = 0.07). Gandhi et al., comparing 24 case-matched patients undergoing right hemicolectomy or anterior rectosigmoidectomy between SILC and hand-assisted laparoscopic colectomy (HALC), reported that the average operative time was longer in SILC as compared to HALC (143 versus 113 min *P* = 0.0004) while there was no difference in conversion rate or perioperative complications [33]. Importantly, average LOS was significantly shorter in the SILC group compared with the HALC group (2.7 versus 3.3 days *P* < 0.02), which was also supported by another case-matched study performing right colectomies where Papaconstantinou et al. [31] reported that LOS was significantly shorter in the SILC group (*n* = 29) compared to LAC (*n* = 29) and HALC (*n* = 29) groups (3.4 versus 4.6 versus 4.9 days, *P* < 0.05). In addition, maximum pain scores on p.o. Days 1 and 2 were significantly lower in the SILC group compared to LAC and HALC groups (*P* < 0.05). On the other hand, in comparison between 16 single-port and 27 conventional laparoscopic right hemicolectomies of similar clinical background, Waters et al. concluded that no significant difference of short-term outcomes was observed between the 2 groups [35]. Adair et al., in their case-matched analysis of 17 single-port and multiport laparoscopic right colectomy cases, also found similar short-term outcomes between the 2 groups [36]. Wolthuis et al., in their case-matched study between SILC (*n* = 14) and LAC (*n* = 14) examining postoperative inflammatory response, reported that C-reactive protein (CRP) levels changed similarly in both groups (*P* = 0.34).

## 4. Discussion

Potential advantages of SILC over other minimally invasive surgeries include a single small skin incision. The length of the skin incision is partly determined by the size of the resected specimen. Extraction difficulties may be encountered with large colon tumors or with obese patients with thick mesentery, omentum, or deep abdominal wall and colon filled with stool. In fact, our paper revealed that the final (at the end of operation) length of incision scar was longer than the initial one in all relevant reports, suggesting that cosmetic analysis on SILC should be based on final, not initial, scar length and objectively based on cosmesis scale or body image scale which has not yet been examined in any literature. In theory, a single midline fascial incision may minimizes trauma to the abdominal muscles, epigastric articles, and parietal nerves made by multiple trocars in LAC cases. This potentially leads to less postoperative pain and long-term additional port site complications; one out of two case-matched studies demonstrated significantly less postoperative pain score in SILC group as compared to LAC and HALS groups although another study failed to show less postoperative use of anesthesia in SILC group.

When introducing any new technology, one significant limitation is often the cost of the procedure. Generally, the initial increases in operative costs associated with laparoscopic techniques are mitigated by reduction in morbidity and duration of hospital stay as a result of the minimally invasive surgery. In fact, several studies which examined both short-term and long-term costs associated with laparoscopic colectomy showed an initial increase in the cost associated with laparoscopic colectomy but a long-term, overall saving. The potential challenge with SILC is that it will require purchase of proprietary instrumentation and additional equipments in some cases which increase overall operative cost. Although potential benefits including fewer conversions, a shorter postoperative recovery or LOS, and less morbidity would make SILC more cost effective, demonstration of any economic benefit over LAC can be difficult. Waters et al. [35] reported that the port itself was purchased at a cost of 550–650 USD compared with average cost of 80 USD of the ports used in the standard LAC cases. The marginal increase in direct operative cost was 310–410 USD per case. With similar operative time and LOS, it can be inferred that the total increase in cost is only that of the port device itself.

Concerning surgical instruments and techniques, SILS has several disadvantages compared with multiport laparoscopic surgery. Standard laparoscopic surgeries are performed through multiports allowing variation of scope placement and angling when met with obstructions. In SILS, no additional ports exist for placement of the scope and maneuvering is greatly restricted by nearby instruments. Therefore SILS requires an experienced surgeon to overcome the difficulties of triangulation, pneumoperitoneum leaks, and instrument crowding. In fact, according to our paper, as many as 9 cases needed to be converted to either open or multiports laparoscopic procedure to get better retraction or aid in colonic mobilization. Some investigators recommend utilizing articulating instruments or since obesity was found to be a common reason for conversion, variable length tools including a bariatric-length bowel grasper or an extra-long laparoscope to minimize external clashing are also recommended [19, 30]. One of the most challenging factors for SILC in attaining widespread use is the additional learning curve required for this technique. The SILC is essentially a one-operating surgeon technique which has a potentially detrimental impact upon resident education, affecting the training of future surgeons as well. Because most surgeons are still performing open colectomy (the prevalence of even standard LAC procedure is still under 25% in the US [44, 45]) or are on their own learning curve for laparoscopy, it requires further analysis to determine the impact that introducing a more technically demanding procedure has on training these surgeons.

## 5. Conclusions

SILC is a challenging procedure but seems to be feasible and safe when performed by surgeons highly skilled in laparoscopy. SILC may have potential benefits over other types of minimally invasive surgeries (LAC or HALC), however this has not yet been objectively shown. In the future, randomized controlled trials with a large number of cases are necessary to determine the role of SILC in cost benefit, cosmetic, and oncologic outcomes.

## Figures and Tables

**Table 1:** Characteristics of patients undergoing single-incision laparoscopic colorectal surgery.

Author/year	No. of patients	Evidence level	Age	Gender (M/F)	ASA (I/II/III/IV)	Past surgical history (%)	BMI (kg/m^2^)	Indication
McNally et al. 2011 [19]	27	3	67^#^ (26–86)	13/14	0/16/9/0	44.4	27^#^	Malignant

Bulut et al. 2011 [20]	10	4	67^#^ (49–83)	2/8	3/6/1/0	60.0	23.5^#^ (20–25)	Cancer

Gaujoux et al. 2011 [21]	13	4	53^#^ (23–82)	5/8	I/II: 13 III/IV: 0	38.5	23.5^#^ (18–30)	Polyp: 5 Crohn: 3 Diverticulitis: 3 Benign: 2

Ramos-Valadez et al. 2011 [22]	20	3	59 (37–76)	11/9	2^#^	50.0	25.9 (20–33)	Benign: 17 Malignant: 3

Katsuno et al. 2011 [23]	31	4	67 (58–79)	14/17	NA	NA	22.5	cancer

Wolthuis et al. 2011 [24]	14	3	56^#^ (30–73)	5/9	0/12/2/0	NA	22 (20–24)	Crohn: 6 Cancer: 3 Adenoma: 3 Diverticulitis: 2

van den Boezem and Sietses 2011 [25]	50	4	65 (21–89)	18/32	NA	22.0	27 (17–35)	Malignant: 31 Diverticulitis: 8 Polyp: 7 Colitis ulcerosa: 4

Gash et al. 2011 [26]	10	4	31^#^ (21–56)	4/6	NA	30.0	22^#^ (20–28)	UC

Champagne et al. 2011 [27]	29	2	61 (25–93)	10/19	NA	27.6	27.4	Cancer: 12 Polyp: 4 Benign: 13

Chew et al. 2011 [28]^#^	21	4	63^#^ (48–63)	13/8	2^#^	NA	NA	Cancer: 14 Polyp: 4 Other: 3

Chew et al. 2011 [28]^##^	11	4	66^#^ (49–80)	5/6	2^#^	NA	NA	Cancer: 10 Polyp: 1

Fichera et al. 2011 [29]	10	4	28 (19–38)	8/2	NA	NA	21.9	UC: 10

Chen et al. 2011 [30]	18	3	69	10/8	I/II: 8 III/IV: 10	NA	23.3^#^ (18–29)	Cancer: 16 Diverticulosis: 2

Papaconstantinou et al. 2011 [31]	29	3	60 (33–87)	13/16	0/16/12/1	34.5	30.0 (23–42)	Cancer: 15 Polyps: 12 Crohn: 2

Ross et al. 2011 [32]	39	4	58 (18–86)	16/23	NA	43.6	25.6 (16–40)	Cancer: 15 Polyps: 12 Diverticulitis: 7 Crohn: 5

Gandhi et al. 2010 [33]	24	3	54	12/12	2.3	41.7	28.5	Benign: 15 Malignant: 9

Keshava et al. 2010 [34]	22	4	67^#^ (18–90)	11/11	NA	NA	27^#^ (19–30)	Cancer: 13 Adenoma: 5 Other: 4

Waters et al. 2010 [35]	16	3	65 (39–82)	8/8	2.5	43.8	29 (20–41)	Cancer: 10 Other: 6

Adair et al. 2010 [36]	17	3	67	5/12	NA	0^#^	26.2	Malignantcy: 11 Polyp: 4 Other: 2

Gash et al. 2010 [37]	20	4	46 (24–81)	7/13	9/5/6/0	40.0	25^#^ (21–37)	Cancer: 8 Crohn: 4 UC: 3 Other: 5

Vestweber et al. 2010 [38]	10	4	64^#^	1/9	2^#^	50.0	26.7^#^	Diverticulitis

Boni et al. 2010 [39]	36	4	69	NA	NA	36.1	NA	Malignant: 32 Polyp: 4

^#^data of right colectomies, ^##^data of anterior resections, ASA: American Society of Anesthesiologist, BMI: body mass index, NA: data not available, UC: ulcerative colitis, SSI: surgical site infection, TME: total mesorectal excision, LAC: multiport laparoscopic colectomy, HALS: hand assisted laparoscopic surgery, UTI: urinary tract infection, ^#^median value.

**Table 2:** Perioperative parameters of single-incision laparoscopic colorectal surgery.

Author/year	Colectomy	Skin incision length (cm)		Operative time	Blood loss	Conversion	Additional port	Mortality	Morbidity	Reoperation
		Initial	Final	(min)	(mL)	(%)	(%)	(%)	(%)	(%)
McNally et al. 2011 [19]	Right sigmoid transverse, and so on.	NA	NA (4–8)	114^#^	50^#^	18.5 (to LAC)	0	0	18.5 (ileus, arrhythmia, etc.)	3.7 (cecal ischemia)

Bulut et al. 2011 [20]	Low anterior resection, and so on.	2.5	NA	229^#^ (185–318)	0^#^ (0–100)	0	20.0	0	20.0 (fluid collection, etc.)	0 (two readmissions)

Gaujoux et al. 2011 [21]	Sigmoid right ileocolonic, and so on.	2.5	3.2^#^ (2.5–5)	150^#^ (100–240)	0^#^ (0–350)	0	0	0	0	0

Ramos-Valadez et al. 2011 [22]	Sigmoid	2.5 or 4	3.3	159	58	5.0 (to LAC)	0	0	10.0 (wound complication)	0

Katsuno et al. 2011 [23]	Sigmoid right	2.5–3	2.7	156 (101–263)	27 (5–60)	0	NA	0	3.2 (wound infection)	NA

Wolthuis et al. 2011 [24]	Right sigmoid	3.5^#^	5^#^ (4–6)	75^#^ (70–105)	0^#^ (0–20)	0	0	0	0	7.1 (negative relaparo-scopy)

van den Boezem and Sietses 2011 [25]	Right sigmoid low anterior resection, and so on.	3	NA (−4.5)	130	NA	4.0 (to LAC)	4.0	0	8.0 (wound infection) 4.0 (hernia) 4.0 (ileus) 2.0 (leakage)	2.0 (anastomotic leakage)

Gash et al. 2011 [26]	Restorative proctocolectomy	2.5	NA	185^#^ (100–381)	NA	0	0	0	10.0 (surgical emphysema) 10.0 (panic attack)	NA

Champagne et al. 2011 [27]	Right left	2.5	3.8	134	NA	10.3 (to open/LAC)	6.9	0	17.2 (ileus, etc.)	0

Chew et al. 2011 [28]^#^	Right	2.5	5^#^ (3–10)	85^#^ (45–150)	NA	4.8 (to LAC)	0	0	4.8 (arrhythmia)	0

Chew et al. 2011 [28]^##^	Anterior resection	2.5	5^#^ (3–7)	120^#^ (65–235)	NA	36.4 (to LAC)	36.4	0	18.2 (leakage, bleed)	0

Fichera et al. 2011 [29]	Total	NA	NA	139 (110–180)	100 (20–400)	0	NA	0	0	0

Chen et al. 2011 [30]	Right	3	4^#^ (3–6)	175^#^ (145–280)	75^#^ (20–700)	16.7 (to open/LAC)	NA	0	16.6 (ileus, wound infection, arrhythmia)	0

Papaconstantinou et al. 2011 [31]	Right	NA	4.5 (2.5–7)	129 (53–187)	60 (20–150)	3.4 (to HALS)	NA	0	3.4 (leakage) 6.9 (SSI) 10.3 (minor wound complication)	3.4 (anastomotic leakage)

Ross et al. 2011 [32]	Right sigmoid ileocolic	NA	4.2 (2.5–8)	120 (68–210)	67 (0–250)	5.1 (to open)	7.7	0	7.7 (wound infection, anastomotic bleeding)	0

Gandhi et al. 2010 [33]	Right rectosigmoid	2.5	3.3 (2–6)	143	63	12.5 (to HALS/LAC)	NA	4.2 (metastatic disease)	8.3 (bleed, wound infection)	0

Keshava et al. 2010 [34]	Right	3	4^#^ (3–6)	105^#^ (85–140)	<100 except two	0	0	0	27.3 (ileus, bleed, wound hematoma)	9.1 (bleed, wound hematoma)

Waters et al. 2010 [35]	Right	2	(2.5–4.5)	106 (71–223)	54 (25–120)	0	0	0	18.8 (wound infection, etc.)	0

Adair et al. 2010 [36]	Right	3	3.8	139 (96–215)	NA	0	11.8	5.9 (pulmonary embolus)	29.4 (ileus, etc.)	NA

Gash et al. 2010 [37]	Right extended right anterior resection (TME), and so on.	2	NA	110^#^ (45–240)	NA	10.0 (to LAC)	0	0	10.0 (ileus) 5.0 (wound infection) 5.0 (bleed) 5.0 (hypertension)	0 (one re-admission)

Vestweber et al. 2010 [38]	Sigmoid	2.5	NA	120^#^ (79–156)	Minimal	10.0 (to open)	10.0	0	10.0 (subcutaneous hematoma)	0

Boni et al. 2010 [39]	Right	3–3.5	2.6^a^ (2.1–3.1)	145 (110–172)	NA	0	NA	0	5.6 (UTI, ileus)	0

^#^data of right colectomies, ^##^data of anterior resections, BMI: body mass index, NA: data not available, UC: ulcerative colitis, SSI: surgical site infection, TME: total mesorectal excision, LAC: multiport laparoscopic colectomy, HALS: hand assisted laparoscopic surgery, UTI: urinary tact infection, ^#^median value, ^a^measured on postoperative day 10.

**Table 3:** Required materials of single-incision laparoscopic colorectal surgery.

Author	Patient's position	Port system		Laparoscope			
		Single port	Trocars	Tip	diameter	Degree	Graspers/scissors
		(diameter, mm)	(diameter, mm)		(mm)	
McNally et al. 2011 [19]	NA	SILS port, Gelport, SSL port	NA	NA	NA	NA	NA

Bulut et al. 2011 [20]	Lloyd-Davis	SILS port	3 trocars (5, 5, 5)	Straight	5	0^°^	5 mm curved endoscopic grasper

Gaujoux et al. 2011 [21]	Modified lithotomy	SILS port	3 trocars (5, 5, 5)	NA	5	0^°^	Standard grasper

Ramos-Valadez et al. 2011 [22]	Modified lithotomy	SILS port, GelPOINT Gelport	3 trocars (5, 5, 5)	NA	5	30^°^	Standard nonarticulated laparoscopic instrumentation

Katsuno et al. 2011 [23]	Lithotomy	Trocar insertion method, SILS port	3 trocars (5, 5, 5 or 12)	Rigid	5	30^°^	NA

Wolthuis et al. 2011 [24]	Supine (right hemicolectomy) Modified Lloyd-Davies (sigmoid resection)	SILS port, Quard Port GelPOINT, SSL access system	3 trocars (5, 5, 5)	NA	5	30^°^	Endo grasp

van den Boezem and Sietses 2011 [25]	Supine (right hemicolectomy) Lithotomy (sigmoid resection)	SILS port	3 trocars (5, 5, 12)	Standard	10	30^°^	Straight atraumatic grasper

Gash et al. 2011 [26]	Dorsolithotomy	SILS port, TriPort	3 trocars (5, 5, 12)	NA	5 or 10	30^°^	NA

Champagne et al. 2011 [27]	NA	SILS port	3 trocars (NA)	NA	NA	NA	NA

Chew et al. 2011 [28]	Supine (rectum: lithotomy)	SILS port, SSL access system, TriPort	3 trocars (5, 5, 12)	NA	NA	NA	NA

Fichera et al. 2011 [29]	Lithotomy	Gelport	4 trocars (5, 5, 5, 12)	Rigid	5	30^°^	NA

Chen et al. 2011 [30]	NA	None^#^	3 trocars (5, 5, 5)	Rigid	5	30^°^	NA

Papaconstantinou et al. 2011 [31]	NA	SILS port	NA	NA	NA	NA	NA

Ross et al. 2011 [32]	Supine	GelPOINT	3 trocars (5, 5, 12)	NA	NA	30^°^	NA

Gandhi et al. 2010 [33]	Supine (rectum: lithotomy)	SILS port, GelPOINT Gelport	3 trocars (5, 5, 5)	NA	5	30^°^	NA

Keshava et al. 2010 [34]	Modified Lloyd Davies	Gelport	4 trocars (5, 5, 12, 12)	NA	10	30^°^	NA

Waters et al. 2010 [35]	NA	SILS port	3 trocars (5, 5, 5)	Rigid	5	30^°^	NA

Adair et al. 2010 [36]	Low lithotomy	SILS port, GelPOINT Gelport, TriPort	3 trocars (NA)	Flexible	5	NA	NA

Gash et al. 2010 [37]	NA	TriPort	3 trocars (5, 5, 12)	NA	5 or 10	30^°^	Johan bowel grasper

Vestweber et al. 2010 [38]	Supine, steep Trendelenburg	SILS port	3 trocars (NA)	NA	5	30^°^	NA

Boni et al. [38]	Supine, left side down, and mild Trendelenberg	SILS port Endocone	3 trocars (NA)	NA	NA	NA	Articulating endograsper

NA: data not available, ^#^surgical glove.

**Table 4:** Postoperative recovery of single-incision laparoscopic colectomy.

Author	Length of specimen	Margins	Dissected lymph nodes	Postoperative analgesia	Time to flatus/bowel movement	Start regular diet	Hospital stay
	(cm)	(% of positive)	(*n*)	(days)	(days)	(days)	(days)
McNally et al. [19]	NA	0	15^#^ (3–32)	NA	NA	NA	3^#^ (2–17)

Bulut et al. [20]	15.3 (10–32)	0	14^#^ (3–20)	NA	NA	NA	7^#^ (4–14)

Gaujoux et al. [21]	NA	NA	NA	NA	(2-3)	1	6^#^ (4–10)

Ramos-Valadez et al. [22]	NA	0	20 in malignant cases	NA	NA	NA	3.2

Katsuno et al. [23]	NA	0	18	1.4 ± 1.2 analgesics times	NA	1.5 + 0.8	9.2

Wolthuis et al. [24]	17^#^ (16–23)	0	12^#^ (8–17)	Total 313 mg (198–650 mg) (levobupivacaine) total 250 *μ*g (158–520 *μ*g) (sufentanyl)	NA	NA	7^#^ (5–9)

van den Boezem and Sietses [25]	NA	0	14 (10-)	NA	NA	NA	6^#^ (3–30)

Gash et al. [26]	NA	NA	NA	NA	NA	36 h^#^ (4–48 h)	3^#^ (2–8)

Champagne et al. [27]	43.5	0	19.4 in malignant cases	NA	NA	NA	3.7

Chew et al. [28] (right hemicolectomy)	18.5^#^ (10.5–34.0)	0	17^#^ (10–30) in malignant cases	NA	NA	NA	6^#^ (5–11)

Chew et al. 2011 [28] (anterior resection)	15.0^#^ (11.0–38.0)	0	14^#^ (6–16) in malignant cases	NA	NA	NA	6^#^ (5–21)

Fichera et al. [29]	NA	NA	NA	NA	1.6 (1–3) ostomy output	3 (2–4)	5.1 (4–7)

Chen et al. [30]	NA	0	19.5^#^ (3–42) in malignant cases	NA 10^#^ (0–60)	2^#^ (1–7)	NA	5^#^ (3–15)
				(Demerol equivalents (mg))			

Papaconstantinou et al. [31]	NA	NA	16.4 (4–38)	NA	NA	NA	3.4 (1–8)

Ross et al. [32]	NA	0	19 (12–39) in malignant cases	NA	2.2 (1–4) 2.9 (1–6)	NA	4.4 (2–8)

Gandhi et al. [33]	NA	NA	24.6 in malignant cases	NA	NA	NA	2.7

Keshava et al. [34]	NA	0	17^#^ (10–23)	NA	NA	NA	5^#^ (3–35)

Waters et al. [35]	18 (14–35)	0	18 (13–22)	NA	NA	NA	5 (2–24)

Adair et al. [36]	NA	NA	20 (12–39)	NA	NA	NA	3.9 + 3.7 (1–18)

Gash et al. [37]	NA	NA	NA	NA (TAP blocks)	NA	4–6 h [7cases] 12–16 h [11cases]	46 h^#^ (8–384 h)

Vestweber et al. [38]	18.5 (15–22)	NA	NA	NA	NA	NA	7^#^ (6–15)

Boni et al. [39]	NA	0	24 (15–29)	NA (regular IV paracetamol infusion)	NA	2	5 (4–14)

NA: data not available, TAP: transvers abdominis plane, ^#^median value.

**Table 5:** Comparison of intraoperative parameters between single-incision laparoscopic colectomy and other minimally invasive surgeries.

Author	Study type	No. of patients	Incision length	Operative time	Blood loss	Conversion (%)
		(groups)	(cm)	(min)	(mL)	
McNally et al. [19]	No case matched	27 versus 46 (SILC versus LAC)	NA	114^#^ versus 135^#^ (*P* = 0.08)	50^#^ versus 50^#^ (*P* = 0.21)	0 versus 13.0 (*P* = NA)

Ramos-Valadez et al. [22]	Case matched	20 versus 20 (SILC versus LAC)	3.3 versus 3.2 (*P* < 0.70)	159 versus 162 (*P* < 0.80)	58 versus 99 **(** ***P*** **<** **0.007)**	0 versus 0

Wolthuis et al. [24]	Case matched	14 versus 14 (SILC versus LAC)	5^#^ versus 5^#^ (*P* = 0.81)	75^#^ versus 83^#^ (*P* = 0.31)	0^#^ versus 10^#^ (*P* = 0.99)	0 versus 0

Champagne et al. [27]	Case matched	29 versus 29 (SILC versus LAC)	3.8 versus 4.5 (*P* = 0.098)	134 versus 104 **(** ***P*** **=** **0.0002)**	NA	17.2 versus 6.9 (*P* = 0.11)

Chen et al. [30]	Case matched	18 versus 21 (SILC versus LAC)	4^#^ versus 4^#^ (*P* = 0.52)	175^#^ versus 165^#^ (*P* = 0.16)	75^#^ versus 50^#^ (*P* = 0.67)	16.7 versus 0 (*P* = 0.052)

Papaconstantinou et al. [31]	Case matched	29 versus 29 versus 29 (SILC versus LAC versus HALS)	4.5 versus 5.1 versus 7.1 **(** ***P*** **<** **0.05)**	129 versus 128 versus 116 (*P* = 0.27)	60 versus 90 versus 71 (*P* = 0.19)	3.4 versus 13.8 versus 13.8 (*P* = 0.20)

Gandhi et al. [33]	Case matched	24 versus 24 (SILC versus HALS)	3.3 versus 6.6 **(** ***P*** **<** **0.00001)**	143 versus 113 (***P*** **=** **0.0004**)	63 versus 91 (*P* = 0.06)	12.5 versus 0 (*P* = 0.083)

Waters et al. [35]	No case matched	16 versus 27 (SILC versus LAC)	NA	106 versus 100 (*P* = 0.64)	54 versus 90 (*P* = 0.07)	0 versus 0

Adair et al. [36]	Case matched	17 versus 17 (SILC versus LAC)	3.8 versus 5.1 (extraction port size)	139 versus 134 (*P* = 0.61)	NA	NA

NA: data not available, SILC: single-incision laparoscopic colectomy, LAC: multiport laparoscopic colectomy, HALS: hand-assisted laparoscopic surgery (colectomy), ^#^median value.

**Table 6:** Comparison of pathological and surgical outcomes between single-incision laparoscopic colectomy and other minimally invasive surgeries.

Author	No. of patients	Margin	Dissected lymph nodes	Length of specimen	Mortality	Morbidity	Readmission	Hospital stay	Postoperative pain score
	(groups)	(% positive)	(*n*)	(cm)	(%)	(%)	(%)	(days)
McNally et al. [19]	27 versus 46 (SILC versus LAC)	0 versus 0	15^#^ versus 17^#^ (*P* = 0.33)	NA	0 versus 4.3 (*P* = NA)	18.5 versus 34.8 (*P*= NA)	NA	3^#^ versus 5^#^ (*P* = 0.07)	NA

Ramos-Valadez et al. [22]	20 versus 20 (SILC versus LAC)	0 versus 0	20.3 versus 18.3 (*P* < 0.68)	NA	0 versus 0	10.0 versus 10.0 (*P* < 1.0)	0 versus 0	3.2 versus 3.8 (*P* < 0.25)	NA

Wolthuis et al. [24]	14 versus 14 (SILC versus LAC)	0 versus 0	12^#^ versus 14^#^ (*P* = NA)	17^#^ versus 18^#^ (*P* = 0.47)	0 versus 0	0 versus 0	0 versus 0	7^#^ versus 6^#^ (*P* = 0.13)	Overall mean1.00 versus 1.39 (*P* = 0.25)

Champagne et al. [27]	29 versus 29 (SILC versus LAC)	0 versus 0	19.4 versus 21.6 (*P* = 0.81)	44 versus 44 (*P* = 0.54)	NA	17.2 versus 24.1 (*P* = 0.28)	NA	3.7 versus 3.9 (*P* = 0.44)	NA

Chen et al. [30]	18 versus 21 (SILC versus LAC)	Distal free margin (cm) 16 versus 13.5 (*P* = 0.094)	19.5^#^ versus 19^#^ (*P* = 0.98)	NA	0 versus 0	16.6 versus 9.5 (*P* = 0.51)	0 versus 0	5^#^ versus 5^#^ (*P* = 0.90)	Demerol usage (mg) 10^#^ versus 10^#^ (*P* = 0.82)

Papaconstantinou et al. [31]	29 versus 29 versus 29 (SILC versus LAC versus HALS)	NA	16.4 versus 16.9 versus 18.1 (*P* = 0.83)	NA	0 versus 0 versus 0	(i) Leakage 3.4 versus 0 versus 0 (*P* = 0.36) (ii) SSI 6.9 versus 10.3 versus 6.9 (*P* = 0.86) (iii) Minor wound complication 10.3 versus 13.8 versus 17.2 (*P* = 0.75)	13.8 versus 6.9 versus 10.3 (*P* = 0.69)	3.4 versus 4.6 versus 4.9 **(** ***P*** **<** **0.05)**	Mean maximum Day 1: 4.7 versus 6.0 versus 6.0 **(** ***P*** **<** **0.05)** Day 2: 3.8 versus 5.2 versus 5.0 **(** ***P*** **<** **0.05)**

Gandhi et al. [33]	24 versus 24 (SILC versus HALS)	NA	24.6 versus 18.6 (*P* = 0.22)	NA	NA	8.3 versus 0 (*P* = 0.15)	NA	2.7 versus 3.3 **(** ***P*** **=** **0.02)**	NA

Waters et al. [35]	16 versus 27 (SILC versus LAC)	0 versus 0	18 versus 16 (*P* = 0.10)	18 versus 18 (*P* = 0.92)	0 versus 3.7 (*P* = 0.44)	18.8 versus 14.8 (*P* = 0.99)	6.3 versus 3.7 (*P* = 0.99)	5 versus 6 (*P* = 0.53)	NA

Adair et al. [36]	17 versus 17 (SILC versus LAC)	NA	20.1 versus 18.6 (*P* = 0.70)	NA	5.9 versus 0 (*P* = NA)	29.4 versus 23.5 (*P* = NA)	NA	3.9 versus 4.1 (*P* = 0.87)	NA

NA: data not available, SILC: single-incision laparoscopic colectomy, LAC: multiport laparoscopic colectomy, HALS: hand-assisted laparoscopic surgery (colectomy).

^#^Median value, SSI: surgical site infection.
